# MukB colocalizes with the *oriC* region and is required for organization of the two *Escherichia coli* chromosome arms into separate cell halves

**DOI:** 10.1111/j.1365-2958.2007.05881.x

**Published:** 2007-09

**Authors:** Olessia Danilova, Rodrigo Reyes-Lamothe, Marina Pinskaya, David Sherratt, Christophe Possoz

**Affiliations:** Department of Biochemistry, University of Oxford Oxford OX1 3QU, UK

## Abstract

The circular *Escherichia coli* chromosome is organized by bidirectional replication into two equal left and right arms (replichores). Each arm occupies a separate cell half, with the origin of replication (*oriC*) at mid-cell. *E. coli* MukBEF belongs to the ubiquitous family of SMC protein complexes that play key roles in chromosome organization and processing. In *mukBEF* mutants, viability is restricted to low temperature with production of anucleate cells, reflecting chromosome segregation defects. We show that in *mukB* mutant cells, the two chromosome arms do not separate into distinct cell halves, but extend from pole to pole with the *oriC* region located at the old pole. Mutations in *topA*, encoding topoisomerase I, do not suppress the aberrant positioning of chromosomal loci in *mukB* cells, despite suppressing the temperature-sensitivity and production of anucleate cells. Furthermore, we show that MukB and the *oriC* region generally colocalize throughout the cell cycle, even when *oriC* localization is aberrant. We propose that MukBEF initiates the normal bidirectional organization of the chromosome from the *oriC* region.

## Introduction

The *Escherichia coli* chromosome adopts a compact structure, the nucleoid, with each locus following a reproducible choreography throughout the cell cycle (reviewed in Espeli and Boccard, 2006). Bidirectional replication, initiating at *oriC* and terminating in the *ter* region opposite to *oriC*, defines two replication arms or replichores. For newborn cells with non-overlapping replication cycles, the two chromosome arms locate to different cell halves, later replicated markers being more pole proximal (Nielsen *et al*., 2006; Wang *et al*., 2006). This Left–Right (<L-R>) chromosome organization is regenerated quickly after replication. However, the mechanisms positioning the sister origins at the cell quarters and/or organizing the two chromosome arms are unknown.

*S*tructural *m*aintenance of *c*hromosomes (SMC) proteins are ubiquitous and required for many aspects of chromosomes segregation in eukaryotes and prokaryotes (Nasmyth and Haering, 2005). SMC dimers adopt a flexible V-shaped structure, whose open ends may come together in a reaction facilitated by partner proteins. Two of the best-studied SMC complexes are cohesins, which hold the sister chromatids together during mitosis, and condensins, which organize mitotic chromosomes. In *E. coli*, as in *Bacillus subtilis*, SMC impairment leads to thermosensitivity, and to the formation of ∼15% anucleate cells at permissive temperature, indicative of a defect in chromosome segregation (Niki *et al*., 1991; Britton *et al*., 1998). These phenotypes are suppressed by a decrease of topoisomerase I activity, while inhibition of gyrase is synthetically lethal with mutation in bacterial SMC (Sawitzke and Austin, 2000; Lindow *et al*., 2002), consistent with MukBEF acting in chromosome organization by organizing DNA supercoiling. The increase of negative supercoiling in *topA* mutants could directly compensate for the impairment of MukBEF organization activity, or the suppression could be an indirect consequence of the change in supercoiling arising as a consequence of TopoI depletion. A direct role of increased negative supercoiling in TopA^–^ in suppressing the Muk^–^ phenotype is supported by data showing that inhibition of gyrase activity reverses this suppression (Sawitzke and Austin, 2000); that negative supercoiling is increased in *E. coli* cells grown at low temperature (Stupina and Wang, 2005), when MukBEF is dispensable; and by the demonstration that MukB constrains DNA in a condensin-like fashion *in vitro* (Petrushenko *et al*., 2006).

Nevertheless, the primary role of bacterial SMC proteins remains elusive (reviewed in Strunnikov, 2006). Here, we have analysed the contribution of MukBEF to <L-R> chromosome organization and have related this to MukB localization in living cells. We show that chromosome organization is changed in *mukB* mutant cells. The two chromosome arms do not separate into distinct cell halves and flank the *ori* region at mid-cell as in Muk^+^ cells, but rather extend from pole to pole with the *oriC* region located at the old pole. Furthermore, we show that MukB and the *oriC* region generally colocalize throughout the cell cycle, even when *oriC* localization is aberrant.

## Results

### Sister origins position aberrantly at opposite poles in *mukB* cells

**Fig. 1:**
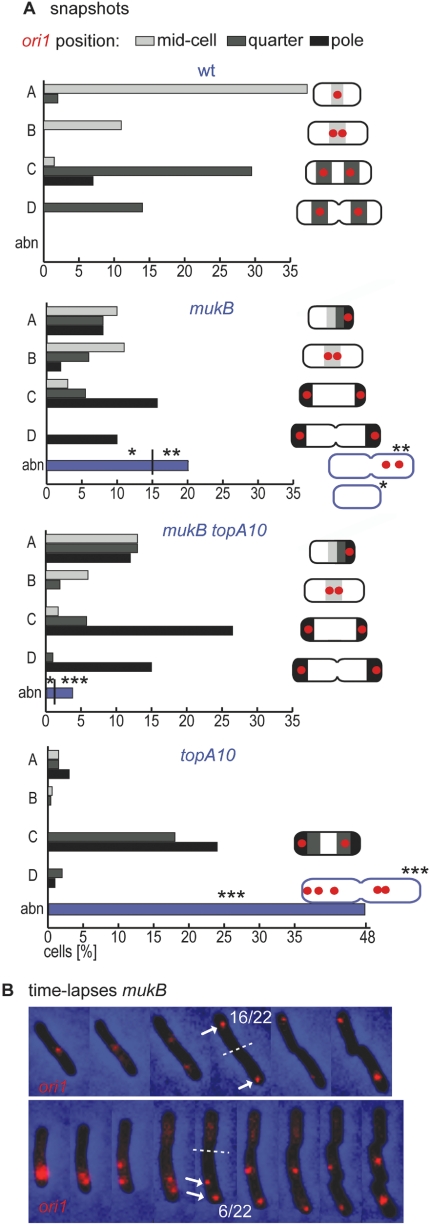
Polar positioning of *ori1* in *mukB* and *mukB topA10* cells. A. Snapshot analysis of *ori1* positioning in different strains as indicated (wt: IL02; *mukB*: OS27; *mukB topA10*: OS47; *topA10*: OS70). For about 500 cells of each strain, cells were first classified into the five types shown in the schematic. Types A–C correspond to non-septating cells with one *ori1* focus (type A), two *ori1* foci closely spaced (type B) and two *ori1* foci well separated (type C); type D corresponds to septating cells with segregated two sister *ori1* foci, and type *abn* corresponds to all the other cells judged abnormal. Cells were divided along the longitudinal axis in six equal parts: left pole, left quarter, mid-cell left, mid-cell right, right quarter and right pole. The *ori1* position (histograms) was classed in either polar (black), quarter (dark grey) or mid-cell (light grey). The predominant position was represented by schematics on the right-hand side of the histograms. Three types of abnormal cells were distinguished: *lacking *ori1*; **containing two *ori1* foci in the same cell half and ***containing more than two *ori1* foci. B. Time-lapse analysis of *ori1* in *mukB* cells. Examples of successful (top) and unsuccessful (bottom) segregation are illustrated. The arrows indicate the position of the sister *ori1* foci at the time of division (dashed line). Images were taken every hour.

Tracking of the *ori1* locus, located 15 kb counterclockwise of *oriC,* by fluorescent repressor-operator sites (FROS; Lau *et al*., 2003), revealed abnormal positioning and segregation of sister origins in *mukB* mutants. In wild-type cells growing at 22°C, without overlapping replication cycles, our snapshot data (Fig. 1A) were consistent with the segregation pathway previously described (Wang *et al*., 2006). *ori1* is always close to mid-cell in newborn cells and after duplication at mid-cell, sister origins migrate in opposite directions towards the quarter positions, where they remain until cell division. In contrast, snapshots of *mukB* mutants showed that duplicated *ori1* foci were most often positioned close to opposite old poles (types C and D; ∼70% of 2×*ori1* cells), with focus duplication being inferred to occur either at an old pole or at mid-cell (type B).

Time-lapse tracking confirmed the snapshot analysis of *mukB* mutants, with all the successful segregations (16/22 cases), showing sister *ori1* segregations to opposite poles (Fig. 1B, top and Fig. S1A and B), rather than the quarter positions observed in wild-type cells. Consistent with this, 21/24 *mukB* newborn cells had *ori1* close to an old pole. Duplication of *ori1* foci occurred either at the pole (3/16; bottom panel) or after movement of a single focus to mid-cell (13/16; Fig. 1B, top). In the cases of unsuccessful *ori1* segregation (6/22 cases), *ori1* failed to duplicate (2 cases, Fig. S1C), and showed delayed *ori1* duplication at a pole (2 cases; Fig. 1B, bottom) or at mid-cell (2 cases). Therefore, when sister *ori1* loci are successfully segregated to *mukB* daughter cells, they position aberrantly close to the old poles. This result suggests that in the absence of MukBEF, chromosome organization is perturbed specifically, with a new type of organization that places newly replicated sister origins at the old poles.

### The *topA10* mutation does not suppress the aberrant *ori* positioning of *mukB* cells

Because impairment of topoisomerase I (*topA10*) suppresses the temperature-sensitivity and anucleate cell production of Muk^–^ cells (Sawitzke and Austin, 2000), *ori1* positioning was analysed in *mukB topA10* mutant cells. As expected, the viability at 37°C in minimal or rich media was restored, and the population contained few anucleate cells (1% as compared with 15% in a *mukB* strain). At 22°C in minimal medium (and at 30°C), most segregated sister *ori1* foci observed in the *mukB topA*10 snapshots showed polar positioning, with a similar pattern to the *mukB* strain (Fig. 1A). Consistent with this, in 15/17 newborn cells observed by time-lapse tracking, *ori1* focus was close to the old pole (not shown). The *topA*10 single mutant cells exhibited a defect in cell division and aberrant *ori1* positioning, apparently as a consequence of impaired chromosome segregation. This phenotype is quite distinct from that of *mukB* and *mukB topA10* cells (Fig. 1A) and has, as its main features, a substantial fraction (48%) of filamentous cells with more than two *ori1* foci (abn), and a corresponding deficiency in cells containing a single *ori1* focus (type A). In cells with two separated sister *ori1* foci, 57% of *ori1* foci were at a pole (but very rarely both together at poles), while the others were at quarter cell, as in wild-type cells. Therefore, the phenotype of *mukB topA*10 cells, when judged by *ori1* positioning, is similar to that of *mukB* cells, despite the fact that temperature-sensitivity and anucleate cell production is abolished in the double mutant. We conclude that the chromosome organization defect of *mukB* cells persists when the *topA10* allele is introduced. Thus, this defect can be functionally separated from temperature-sensitivity and anucleate cell production.

### Left–Right (<L-*ori*-R>) nucleoid organization is perturbed in *mukB* cells

**Fig. 2:**
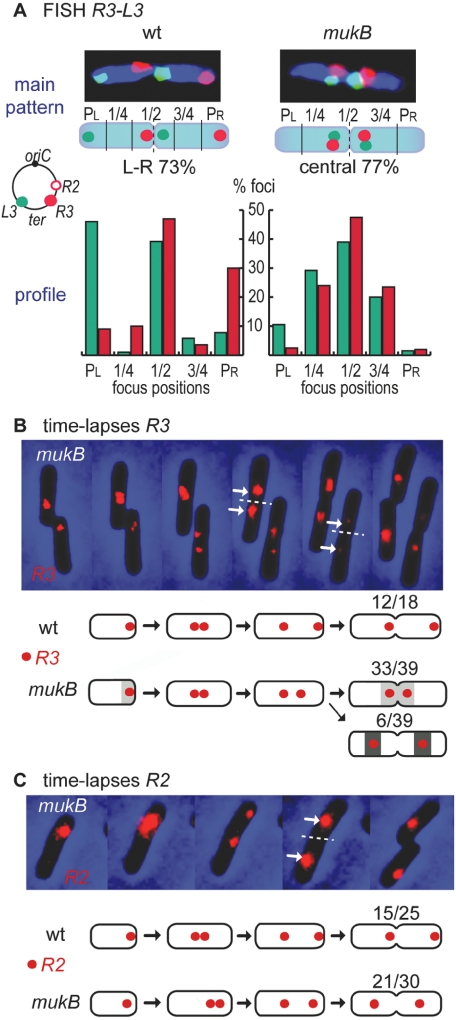
Perturbation of <L-R> nucleoid organization in *mukB* cells. A. *L3* and *R3* positions by two-colour FISH. Analysis was restricted to four-foci cells having a normal nucleoid (as judged by DAPI staining). Foci from 123 MukB^+^ cells (AB1157) and 150 *mukB* cells (OS53) were binned into five positions from pole left (P_L_) to pole right (P_R_). The predominant four-focus patterns and the *L3–R3* profiles are shown. B. Time-lapse analysis of *R3* by FROS. Images of *mukB* cells (OS55) were taken every hour, kept at room temperature (G ∼5.5 h, as judged by cell elongation). Below, schematics show the predominant segregation pathways of *R3* loci in wild-type and *mukB* cells. C. Same as B for *R2* locus (OS30).

The above results show that the <L-*ori*-R> organization about the transverse axis of normal cells is altered when MukBEF is absent, with polar *ori* positioning being reminiscent of the type of organization in *Caulobacter crescentus* (Viollier *et al*., 2004) and in chromosome 1 of *Vibrio cholera* (Fogel and Waldor, 2005). To further probe nucleoid organization, *mukB* cells grown at 22°C were examined using two-colour fluorescent *in situ* hybridization (FISH) with probes to *L3* and *R3* chromosome loci located on opposite arms at positions 2269 and 872 kb respectively close to the *ter* region (Fig. 2A). The patterns of *L3–R3* positioning (Fig. 2A, top), and the *L3* and *R3* population profiles (bottom), were analysed in cells containing duplicated foci for each locus. These four-foci cells were enriched for by the addition of the cell division inhibitor cephalexin for 2.5 h (one-third of a generation time) prior to analysis (Wang *et al*., 2006). A <L-R> pattern in each sister nucleoid, arranged as <*L3–R3–L3–R3*> in pairs of sister nucleoids, was predominant (73%) for the wild-type strain, as reported earlier (Wang *et al*., 2006). This wild-type organization is also revealed by the *L3–R3* population profile, in which L3 peaks at the left pole (P_L_) and at mid-cell, while *R3* peaks at mid-cell and at the right pole (P_R_).

The <L-R> organization about a transverse axis was absent in *mukB* mutants. Instead, the two pairs of sister foci cluster in the central region of the cell close to the newly developing poles (77% of nucleoid pairs; also see the population profile). This result was confirmed by time-lapse tracking of the *R3* locus using FROS in cells growing at room temperature (∼22°C). In the wild-type cells, segregation gives rise predominantly to asymmetric positioning of sister *R3* (or *R2*) loci with respect to mid-cell, generating one cell with *R3* close to the old pole and one cell with *R3* close to the new pole (cartoons, Fig. 2B; Wang *et al*., 2006). In *mukB* mutants, the segregation led to a symmetrical pattern of sister *R3* foci remaining in the central region, producing two daughter cells with a new pole-proximal *R3* locus (time-lapse and cartoons, Fig. 2B and Fig. S2). Consistent with this, a symmetrical pattern about the cell quarter positions of sister *R2* loci was observed in the *mukB* mutant (Fig. 2C). Therefore, the positioning patterns of *R2*, *R3* and *L3* loci suggest an absence of <L-R> organization about a transverse cell axis in *mukB* cells. The results are consistent with <L-R> chromosome organization having moved from being arranged about a transverse axis to apparently being organized about a longitudinal axis, with the two arms extending from the old pole to the new pole, with or without twisting about each other. However, proof of this would require a very extensive analysis with many pairs of loci analysed.

### MukB clusters localize to the origin region

**Fig. 3:**
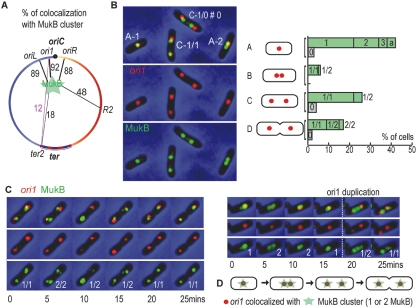
Colocalization of MukB with the origin region. A. Colocalization is indicated as a number (%) on black lines linking MukB and each given loci positioned on the circular map of the *E. coli* chromosome (*ori1*, *R2*, *ter2* loci from OS18 and OS29, OS69, OS19 cells respectively). Colocalization (%) of *ter2* with *ori1* in IL05 cells is indicated on the purple line. B. Snapshot analysis of MukB-GFP and *ori1* positioning. Images of representative wild-type cells (OS18; see histogram for legend). Histogram representing the proportion of the different MukB patterns for each cell type (A–D, Fig. 2). Green bars correspond to MukB/*ori1* colocalization, and patterns 1, 2 and 3 correspond to one, two or three MukB foci colocalizing with one *ori1* focus. When cells contains two *ori1* foci, each *ori1* focus can colocalize either with one or two MukB, generating pattern 1/1, 1/2 or 2/2. Grey bars and pattern 0 correspond to the absence of colocalization of one *ori1* focus with MukB, including cells containing one *ori1* focus or two *ori1* foci (patterns 0/0, 0/1 and 0/2 are included in pattern 0). Pattern ‘a’ corresponds to cells with an extra MukB focus distant from the *ori1*/MukB colocalizing foci. C. Time-lapse tracking simultaneously MukB and *ori1* (without or with *ori1* duplication, left and right respectively; OS18 cells). The number of MukB foci per cluster (bottom panel) and the time intervals between images (below panels) are indicated. D. Representation of the cell-cycle dynamics of *ori1* and MukB deduced from snapshot and time-lapse analysis.

Knowing that MukBEF forms foci within cells, when assayed by immunocytochemistry (den Blaauwen *et al*., 2001), or with a fluorescent fusion protein (Ohsumi *et al*., 2001), we analysed their position and dynamics with respect to chromosomal loci. For simultaneous visualization, the chromosomal *mukB* gene was replaced by the functional *mukB-gfp4* fusion gene, and FROS in cells grown at 30°C was used to track *ori1* (3908 kb), *oriL* (3713 kb), *oriR* (4139 kb), *R2* (366 kb) or *ter2* (1801 kb) loci (Fig. 3A).

MukB-GFP foci appeared either as a single focus or as two (and sometimes three) closely spaced foci (MukBEF clusters); interconversion between these states was reversible and dynamic (Fig. 3C). Thus, we have chosen to score the frequency of colocalization (%) of a given genetic locus with a MukB cluster, by examining at least 400 cells for each locus. A genetic locus is defined as colocalizing with a MukB cluster if there is an overlap between the genetic locus focus and a MukB focus (that may belong to a cluster) when images were overlaid.

For a region of at least 400 kb centred on *oriC*, the colocalization of *ori* loci with MukB clusters was greater than 88%, revealing a preferential colocalization with the origin region. Increasing the distance from *oriC* reduced gradually the colocalization with a MukB cluster (48% for *R2* and 18% for *ter2*). Because the *ter* region crosses the *ori* region during each replication cycle (Wang *et al*., 2006), it is not surprising that we observe a low, but significant, colocalization of *ter2* with both *ori1* (purple line) and MukB. Within the cell population, the ratio of MukB foci to *ori1* foci was 1.4. Different MukB/*ori1* patterns were observed, but predominantly (83% of cells) each *ori1* is colocalizated with one or two MukB foci (patterns 1 and 2 in cells with one origin; and 1/1, 1/2 and 2/2 in cells with two origins, Fig. 3B). Time-lapse tracking revealed that the MukB-GFP signal could reversibly interconvert between one and two MukB foci within a 5 min time frame, in situations where *ori1* duplication did not occur (12/13 cases; Fig. 3C left and Fig. S3A). The duplication of MukB was observed either concomitantly (within 5 min, the time between two images) with *ori1* (4/9 cases, Fig. 3C, right and Fig. S3B) or 5 min before (5/9 cases, not shown). The MukB cluster could interconvert within these intervals. Thus, dynamic MukB clusters tend to colocalized with *ori1* throughout the cell cycle (Fig. 3D).

### Mispositioned or non-duplicating origin regions remain colocalized with MukB

**Fig. 4:**
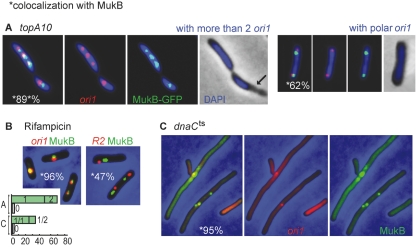
Mispositioned or non-duplicating *ori1* foci colocalize with MukB. A. *topA10* cells (OS70) containing more than two *ori1* (left) or containing a polar *ori1* (right). B. Wild-type cells after 3 h of rifampicin treatment with *tetO* array inserted at either position *ori1* (left, OS29 cells; see Fig. 3 for the histogram legend) or *R2* (right, OS69 cells). C. *dnaC*^ts^ cells (OS82) grown at 37°C for 8 h with *tetO* at *ori1.* The percentages in panels A, B and C refer to the colocalization of *ori1* or *R2* loci with MukB clusters, as deduced from the examination of at least 400 cells.

In order to test whether MukB positioning depends on *ori1* location, the relative MukB and *ori1* positioning were analysed in *topA10* mutant cells grown at 30°C, where aberrant origin positioning occurs (Fig. 1A and 35% of origins are positioned at a nucleoid edge). Colocalization of a polar *ori1* focus with a MukB cluster was substantial in normal-length cells (62%, Fig. 4A). Furthermore, the colocalization was maintained (89%) in the 48% of filamentous cells containing more than two *ori1* foci (Fig. 1).

Furthermore, the colocalization of *ori1* and MukB was not affected when cells contained completely replicated chromosomes and were unable to re-initiate replication, by using rifampicin or a *dnaC*^ts^ mutant (Fig. 4B and C). After 3 h of rifampicin treatment at 30°C, the MukB/*ori1* ratio is close to 1, most MukB clusters being replaced by a single focus (compare Figs 3B and 4B left). The colocalization of R2, an *ori*-distal locus, with MukB was not increased after rifampicin treatment (Fig. 4B right). DnaC impairment for 8 h did not lead to loss of *ori1* colocalization with MukBEF, although now there were additional MukBEF foci distant from *ori1*, but still colocalized with the nucleoid. The view that MukB focus formation requires DNA association is supported by the observation that out of ∼500 cells lacking an *ori1* focus (> 90% of which do not exhibit any DAPI staining; data not shown), which were observed after DnaC impairment, none exhibited a MukB focus (two such cells without *ori1* are shown in Fig. 4C). Therefore, the origin regions remained predominantly colocalized with MukBEF clusters during extensive periods of DNA replication arrest.

## Discussion

We have revealed that in a *mukB* mutant, the two chromosome arms are not separated into distinct cell halves and the sister origins lose their normal positioning, migrating to the outside poles after duplication. A perturbation of the *oriC* region in a *mukB* mutant was reported by Weitao *et al*. (2000), but probably because of their use of faster growth conditions and the absence of time-lapse analysis, they concluded that *oriC* positioning was random rather than polar. The suppression of the thermosensitivity and of the formation of anucleated cells by topA10 mutation led to the model that MukB action, within a MukBEF complex, is limited to the organization of DNA supercoiling (Sawitzke and Austin, 2000). However, we observed that the aberrant *ori1* positioning is not suppressed by a topA10 mutation. Thus, one interpretation that we favour is that the action of MukB involves more than the organization of DNA supercoils. Moreover, the absence of suppression in a strain where the indirect consequences of MukB loss should be reduced (as *mukB topA10* double mutants have a normal viability) suggests that the aberrant positioning of the origin is a direct consequence of MukB loss.

In addition, we have shown the preferential colocalization of MukBEF with the *ori* region, with one, two or three MukB foci being apparently associated with a given *ori* region, interconversion of focus number being frequent. Previous work, using either immunocytochemistry (den Blaauwen *et al*., 2001) or fluorescent protein fusions (Ohsumi *et al*., 2001), has shown that MukBEF forms foci or clusters but did not track MuBEF position in relation to specific genetic markers, and did not use time-lapse analysis. Consequently, they did not report an apparent association with the *ori* region and the rapid interconversion of focus number associated with a given *ori*. Nevertheless, the results of Ohsumi *et al*. (2001) appear consistent with those reported here. We propose that the MukBEF complex acts in the origin region to initiate bidirectional organization after replication.

**Fig. 5:**
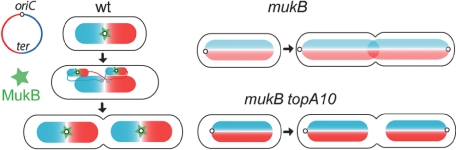
Model of *E. coli* chromosome organization/segregation. Wild-type: Chromosomes segregation is facilitated by the <L-R> organization initiated by MukB colocalizing with the *ori* regions. *mukB* (22°C) and *mukB topA10*: ‘MukB-free’ segregation is allowed only when the level of negative DNA supercoiling is increased (low temperature/*topA10*) but leads to an aberrant arrangement, with the two arms extending (twisted or not with each other) from the old poles to the new poles. For clarity, the two replichores are represented as untwisted. The chromosome organization in *mukB topA10* cells is extrapolated from the aberrant origin positioning.

We consider three types of mechanism that could give rise to the observations of MukB-*ori* colocalization. The first involves a direct interaction of MukBEF with some feature of the *ori* region (Fig. 5). This could relate to some sequence motif(s) present in the *ori* region, or to some other aspect of *ori* biology. Alternatively, the *ori* region and MukBEF could be targeted by independent mechanisms to the same cellular compartment. This seems unlikely, given that MukB clusters still colocalize with *ori1* when it is positioned aberrantly. A third possible mechanism is a rosette model in which MukBEF molecules bind discrete sites spread over the chromosome (for example, one per topological domain), and MukBEF–MukBEF interactions lead to a rosette-like structure, centred on the origin region.

At present we favour the first mechanism, although we have failed to show a preferential association of MukBEF with *ori* sequences using chromatin immunoprecipitation assays that employed either real-time polymerase chain reaction (PCR) or microarrays assays. Indeed, in two independent microarray experiments, DNA fragments enriched by the co-immunoprecipition of MukB were not exclusively contained in the origin region, but scattered over the whole chromosome (M. Pinskaya *et al*., unpubl. data). There is no evidence that any SMC complex can recognize specific DNA sequences directly. Nevertheless, both cohesins (Nasmyth and Haering, 2005) and condensins (Wang *et al*., 2005a) can associate with specific chromosome regions. Such localization may reflect places of DNA loading and/or positions to which the SMC complex is directed [for example by transcription (Lengronne *et al*., 2004) or by other protein–DNA complexes]. In *E. coli,* such an association seems to occur within the ‘Ori macrodomain’, a ∼1 Mbp region that behaves as a single unit in some assays (Espeli and Boccard, 2006), but wherein loci can still behave independently (Fekete and Chattoraj, 2005).

The <*ori*-out *ter*-in> chromosome arrangement proposed for *mukB* cells (Fig. 5) is similar to that observed for *C. crescentus* and chromosome 1 of *V. cholera,* although both express functional SMCs, and the origins are actively transported to the poles, which could force structuring of the two chromosome arms about a polar origin (Viollier *et al*., 2004; Fogel and Waldor, 2005). Consistent with this, when the *V. cholera* chromosome I transport system is mutated, the origins adopt similar positions to *E. coli* origins (Fogel and Waldor, 2006), suggesting that the chromosome I of *V. cholerae* would also have a <L-R> organization in absence of constraint on its origin positioning. Jun and Mulder (2006) have shown how entropy considerations alone may direct a given pattern of bacterial chromosome organization and segregation. Such a pattern may be modified by proteins that transport DNA, or which organize specific chromosomal regions. Our model of MukBEF action at sister *ori* regions is consistent with the positioning of *ori* at mid-cell as a consequence of space restriction: within a confined rod-shaped cell, an equal amount of chromosome DNA is placed around the left and the right *ori* sides. Thus, the organization of the two chromosome arms via MukBEF would facilitate the segregation of the sister chromosomes without the need to position any chromosome region along the cell.

## Experimental procedures

The bacterial strains used are listed in Table S1. *E. coli* AB1157 strains containing *tetO* or *lacO* arrays were constructed as previously described (Wang *et al*., 2005b). We were unable to construct stable *mukB* strains containing both L3 and R3 operator arrays when the cognate repressors were present. TetR-CFP and LacI-CFP were expressed from pWX9 and pWX17 plasmids respectively, and both TetR-YFP and LacI-CFP from pWX6 plasmid (Wang *et al*., 2005b). To prevent replication blockage (Wang *et al*., 2006), AT (40 ng ml^−1^) and IPTG (0.5 mM) were added to the culture of strain containing the *tetO*/TetR-CFP system and the *lacO*/LacI-CFP system respectively. *tetO*/TetR-CFP was used for all FROS visualization in *topA10* and *mukB* single and double mutants and the parental strain. *tetO*/TetR-CFP and *lacO*/LacI-CFP were both used for FROS visualization simultaneously with MukB-GFP. *mukB-gfp*4 (Ohsumi *et al*., 2001), *mukB::km* (Niki *et al*., 1991) and *dna*C2 (Zhou *et al*., 1997) alleles were introduced by transduction in AB1157 derivatives. *topA10* allele (Biek and Cohen, 1989) was transduced to AB1157 *mukB::km* selecting transductants at 37°C, and then *mukB-gfp* allele was transduced. For snapshot experiments, except when indicated otherwise, cells were cultivated in M9 sodium-acetate supplemented with 100 μg ml^−1^ arginine, histidine, leucine, threonine and proline. For time-lapse experiments, cells were grown on a microscope slide coated with an agarose layer prepared with the medium used for snapshot experiment. The doubling time in liquid was ∼8 h at 22°C for MukB^+^ and *mukB*::*km* strains, and ∼4.8 h at 30°C for MukB^+^ cells, ensuring that newborn cells contain a non-replicating chromosome, as confirmed by flow cytometry (not shown). Microscopy using FROS and FISH was performed as described by Wang *et al*. (2006). Chromatin immunoprecipitation were realized with little modifications (see *Supplementary material*) to the protocol described previously (Kuras and Struhl, 1999) with *E. coli* AB1157 cells expressing MukB-GFP as the only MukB source, using anti-GFP antibodies (Roche). Microarrays and DNA hybridizations were realized by Oxford Gene Technology company (http://www.ogt.co.uk).

## Supplementary material

The following supplementary material is available for this article:

Fig. S1 Time-lapse analysis of *ori1* in *mukB* cells (OS27).

Fig. S2 Additional time-lapse analysis of *R3* by FROS in *mukB* cells (OS55).

Fig. S3 Time-lapse tracking simultaneously MukB and *ori1* [without (A) or with (B) *ori1* duplication].

Table S1 Bacterial strains.

This material is available as part of the online article from: http://www.blackwell-synergy.com/doi/abs/10.1111/j.1365-2958.2007.05881.x (This link will take you to the article abstract).
