# Supplementary material for: MukB colocalizes with the oriC region and is required for organization of the two Escherichia coli chromosome arms into separate cell halves
Source: Mol Microbiol. 2007 Sep;65(6):1485–92. doi: 10.1111/j.1365-2958.2007.05881.x (PMC2169520; doi:10.1111/j.1365-2958.2007.05881.x)
Supplement: Fig. S1 — Time-lapse analysis of ori1 in mukB cells (OS27). [file mmi0065-1485-SD1.pdf]

Supplementary data for the article of Olessia Danilova, Rodrigo Reyes-Lamothe, Marina Pinskaya, David Sherratt[1] and Christophe Possoz entitled "MukB co-localises with the *oriC* region and is required for organisation of the two *Escherichia coli* chromosome arms into separate cell halves".

[1]Department of Biochemistry, University of Oxford, Oxford OX1 3QU, United Kingdom. Co-corresponding authors: D.J. Sherratt (Sherratt@bioch.ox.ac.uk) and C. Possoz (possoz@cgm.cnrs-gif.fr).

Fig. S1. Time-lapse analysis of *ori1* in *mukB* cells (OS27). Other examples of successful (A and B) and unsuccessful (C) segregation are illustrated. The arrows indicate the position of the sister *ori1* at the time of division (dashed line). Images were taken every hour.

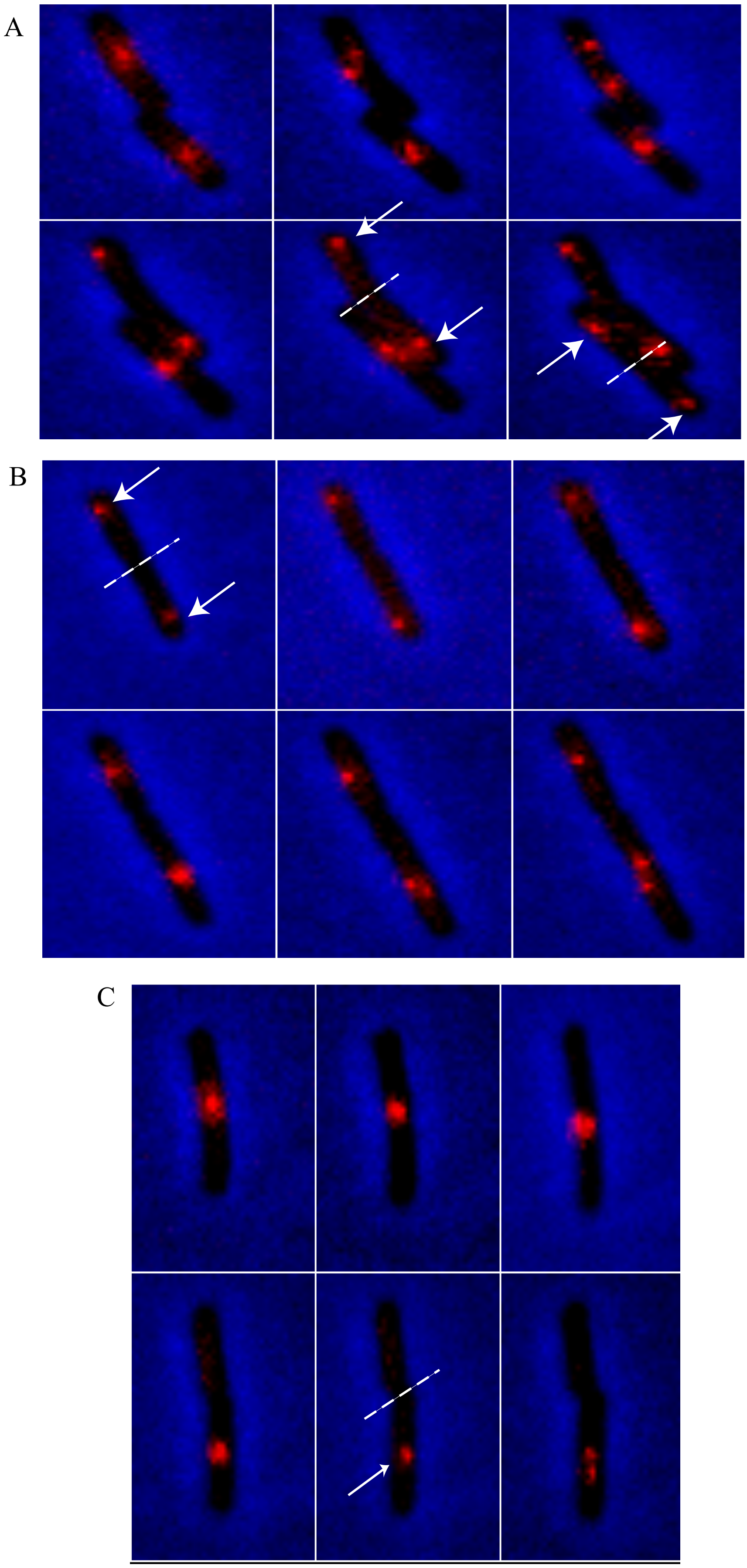

Fig. S2. Additional time-lapse analysis of *R3* by FROS in *mukB* cells (OS55). Images of were taken every hour, kept at room temperature.

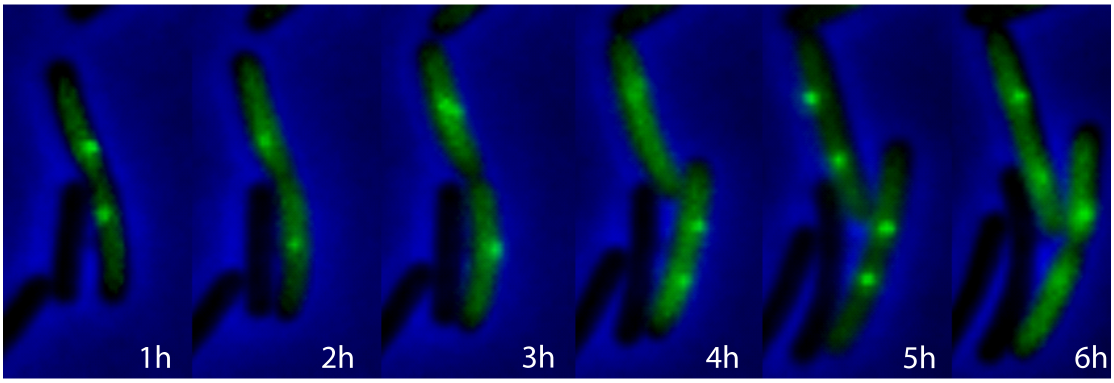

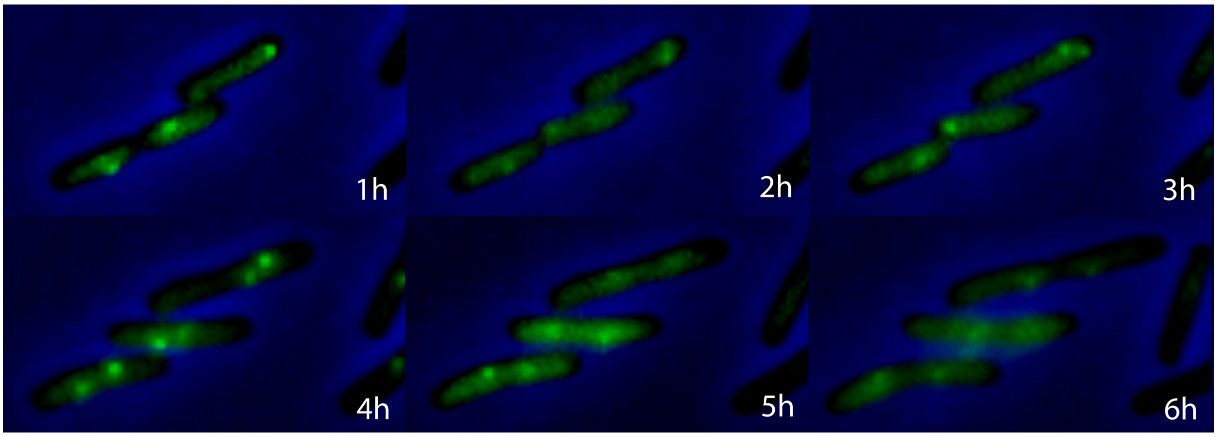

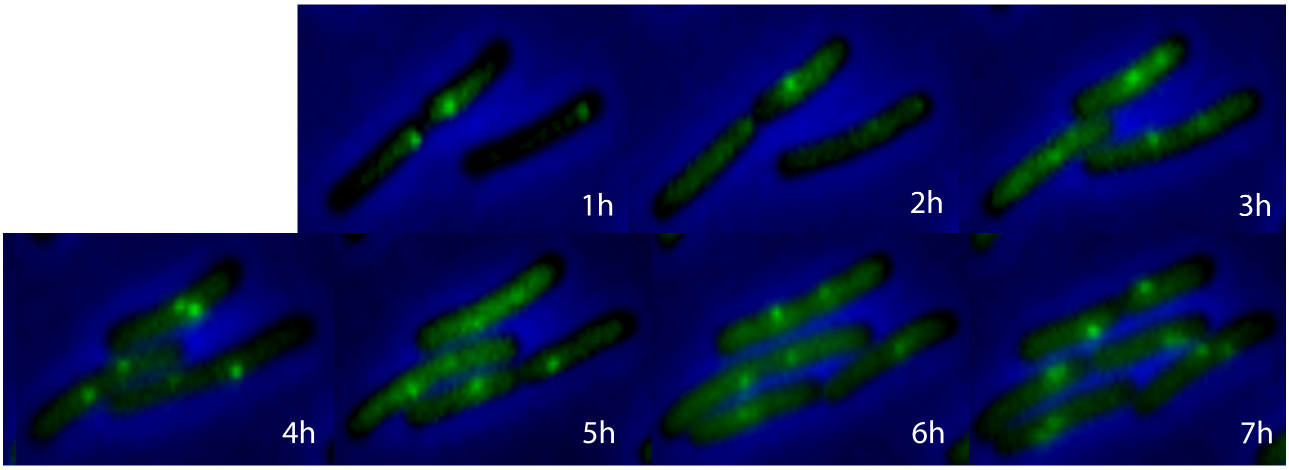

Fig. S3. Time-lapse tracking simultaneously MukB and *ori1* (without (A) or with (B) *ori1* duplication). Images of OS18 cells growing at 32°C were taken every 5 mins (Top: overlay images; middle : *ori1* positions and bottom: MukB positions).

A.

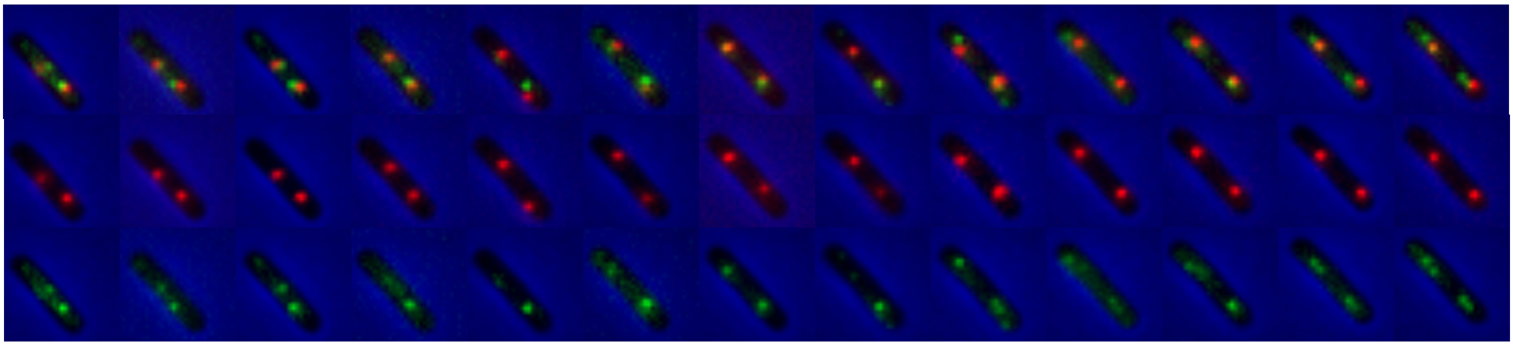

B. *ori1* duplication

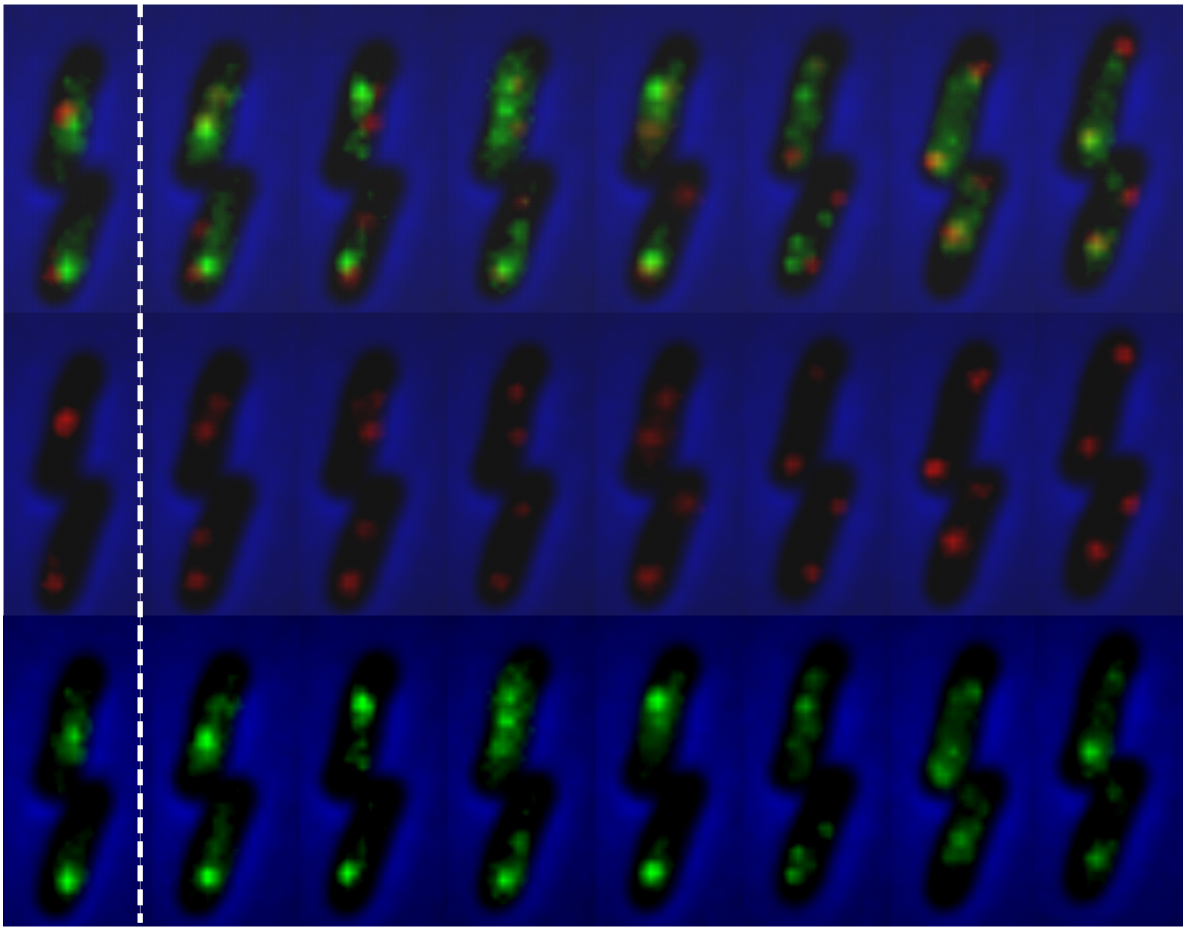

Table S1: Bacterial strains

Name	Relevant genotype	Reference
KAT1	*mukB-gfp*4	Ohsumi et al., 2001
GC7528	Δ*mukB::km*	Niki et al., 1991
DPB923	*topA10*	Biek and Cohen, 1989
AB1157	*F– thr-1 leuB6 proA2 his4 thi1 argE3 lacY1galK2 rpsL supE44 ara-14 xyl-15 mtl-1, txs-33*	Bachmann, B.J. (1987) *in Escherichia coli* and *Salmonella typhimurium*: Cellular and Mol. Biology (Neidhard,et al; eds.) vol. 2, pp. 1190-1219, ASM, Washington D.C.
IL02	AB1157 $tetO_{240}$ @ *ori1*	Lau et al., 2003
IL05	AB1157 $tetO_{240}$ @ *ori1,* $lacO_{240}$ @ *ter2*	Lau et al., 2003
OS18	AB1157 $lacO_{240}$ @ *ori1*, *mukB-gfp*4	This work
OS29	AB1157 $tetO_{240}$ @ *ori1*, *mukB-gfp*4	This work
OS82	AB1157 $tetO_{240}$ @ *ori1*, *mukB-gfp*4, *dna*C2	This work
OS69	AB1157 $tetO_{240}$ @ *R2*, *mukB-gfp*4	This work
OS19	AB1157 $lacO_{240}$ @ *ter2*, *mukB-gfp*4	This work
OS53	AB1157 Δ*mukB ::km*	This work
OS27	AB1157 $tetO_{240}$ @ *ori1*, Δ*mukB ::km*	This work
OS30	AB1157 $tetO_{240}$ @ R2, Δ*mukB ::km*	This work
OS55	AB1157 $tetO_{240}$ @ R3, Δ*mukB ::km*	This work
OS47	AB1157 $tetO_{240}$ @ *ori1*, Δ*mukB ::km* *topA10*	This work
OS70	AB1157 $tetO_{240}$ @ *ori1*, *mukB-gfp*4 *topA10*	This work

Supplementary Materials and Methods:

Chromatin immunoprecipitation (ChIP) was performed as described (Kuras L. Characterization of protein-DNA association *in vivo* by chromatin immunoprecipitation. Methods Mol Biol. 2004;284:147-62) with the following modifications. Cells were grown in 300 ml of Luria–Bertani (LB) medium at 30°C. Proteins were crosslinked to DNA during the logarithmic growth phase ($1.5x10^7$ cells/ml) at room temperature for 30 minutes and then subjected to sonication at 4^0C to achieve 500 bp DNA fragmentation. Sonicated lysate was clarified by centrifugation at 13,000 rpm for 10 min. Immunoprecipitation was then performed using a rabbit polyclonal antibody against GFP (Invitrogen) and protein A Sepharose beads (Amersham). Immunoprecipitated DNA samples and total DNA samples were purified using Qiagen PCR purification kit and send for microarray analysis to Oxford Gene Technology (Grainger D.C., Hurd D., Harrison M., Holdstock J., Busby S.J. Studies of the distribution of *Escherichia coli* cAMP-receptor protein and RNA polymerase along the *E. coli chromosome*. *Proc. Natl Acad. Sci.USA.* 2005;**102**:17693–17698).
